# Multi-color lasing in chemically open droplet cavities

**DOI:** 10.1038/s41598-018-32596-8

**Published:** 2018-09-20

**Authors:** Lu Zheng, Min Zhi, Yinthai Chan, Saif A. Khan

**Affiliations:** 1 Department of Chemical and Biomolecular Engineering, 3 Engineering Drive 3, National University of Singapore, Singapore, 117582 Singapore; 2 Department of Chemistry, 3 Science Drive 3, National University of Singapore, Singapore, 117543 Singapore

## Abstract

In this paper, we demonstrate FRET-based multicolor lasing within chemically open droplet cavities that allow online modulation of the gain medium composition. To do this, we generated monodisperse microfluidic droplets loaded with coumarin 102 (donor), where the spherical droplets acted as whispering gallery mode (WGM) optical cavities in which coumarin 102 lasing (~ 470 nm) was observed. The lasing color was switched from blue to orange by the introduction of a second dye (acceptor, rhodamine 6 G) into the flowing droplet cavities; subsequent lasing from rhodamine 6 G (~ 590 nm) was observed together with the complete absence of coumarin 102 emission. The ability to control color switching online within the same droplet cavity enables sequential detection of multiple target molecules within or around the cavity. As a demonstration of this concept, we show how the presence of FITC-Dextran and methylene blue (MB) in the medium surrounding the lasing droplets can be sequentially detected by the blue and orange laser respectively. The method is simple and can be extended to a range of water-soluble dyes, thus enabling a wide spectral range for the lasing with the use of a single pump laser source.

## Introduction

Optofluidic dye lasers, in which optical cavities are integrated into microfluidic devices, enable high sensitivity detection of analytes in small volume and low concentration samples^1^. Online color switching of optofluidic dye lasers is useful in a wide variety of fields, from on-chip spectroscopy to flow cytometry, photochemistry, and molecular diagnostics^2–7^, where multiple lasing frequencies are necessary for the excitation of different fluorescent labels^5^. Multi-color dye lasers employing Fabry-Pérot cavities or ring resonators in conjunction with microfluidic channels have been demonstrated^8–10^. The use of passive resonators (i.e. a cavity with reflecting surfaces containing a homogeneous, isotropic and passive dielectric medium but no active gain medium) in these systems requires a tapered fibers or prisms for coupling light into and out of the cavity, which limits their practicality. Active resonators containing a gain medium, where remote excitation and collection of fluorescence spectra can be achieved, have emerged as an alternative to passive resonators^11^. Microfluidic droplets are effective optical microcavities due to their small sizes and surfaced tension-smoothened spherical surfaces^12^. Optical whispering gallery modes (WGMs) can circulate along the droplet surface and provide optical feedback for the gain medium dissolved in the droplet to lase^13^. Lasing action from dye-containing microfluidic droplets suspended in an immiscible fluid with a lower refractive index has been demonstrated^5,12,14^. In a recent work, we have demonstrated the incorporation of dye-based liquid lasers within or around flowing aqueous microfluidic droplets, which enables WGM-based ‘on drop’ sensing^15^. Online tuning of lasing wavelength from microfluidic droplet cavities has also been demonstrated^5,12^. Tang *et al*.^5^. have demonstrated a fast-switching multi-color droplet dye laser by alternatively generating droplets containing different dye molecules, which can be excited by the same pulsed pump laser. While enabling high frequency color switching (up to 100 kHz), the limited choice of dyes that can be directly excited by the same pump laser constrains the wavelength tuning range (between 580 to 680 nm) in this method. In a subsequent work, Tang *et al*.^12^. demonstrated continuous wavelength tuning by tuning the size of droplets online as they flow through a microfluidic chip; this method also has a limited tuning range (700 to 620 nm), within the fluorescence emission spectrum of the dye molecule used. Furthermore, decreasing the droplet cavity diameter also reduces its quality factor, making it harder for the droplets to lase^12^.

In the aforementioned microfluidic droplet lasers, the dyes are excited by tuning a pump laser directly into their absorption bands. As a result, the selection of the dyes is significantly limited by the wavelength of the chosen pump laser^16^. Alternatively, the dyes can be excited indirectly through an energy transfer process, where acceptor lasing is achieved via energy provided by a donor molecule, which is in turn excited by a pump laser^3,17–23^; however, this has not been demonstrated for microfluidic droplet cavities. Research in the field of optofluidics has hitherto exploited the microfluidic droplets as cavities that confine light (via WGMs) and as compartments that confine matter. In this work, we exploit another feature of droplet-based cavities – that they are *chemically open* systems that allow molecular *exchange*. Here, we present the first demonstration of multi-color lasing within chemically open droplet cavities via the online introduction of a second gain medium into the droplets followed by a non-radiative Förster energy transfer (FRET) process. Specifically, we demonstrate this by introducing a dye (acceptor, rhodamine 6 G) from the continuous phase into droplet cavities that initially contain a dye (donor, coumarin 102). We also demonstrate a simple proof-of-concept application of sequential detection of two analytes (FITC-Dextran and methylene blue (MB)) enabled by online laser color switching. While FITC-Dextran is detected before laser color switching, MB is detected only after the switch of laser color. Owing to the FRET pumping mechanism and chemically open droplet cavities, our method can be extended to a wide selection of water-soluble laser dyes, where an extended laser color switching range can be achieved via sequential energy transfer, instead of dyes being excited directly by a single pump pulse laser.

## Results and Discussion

**Figure 1:**
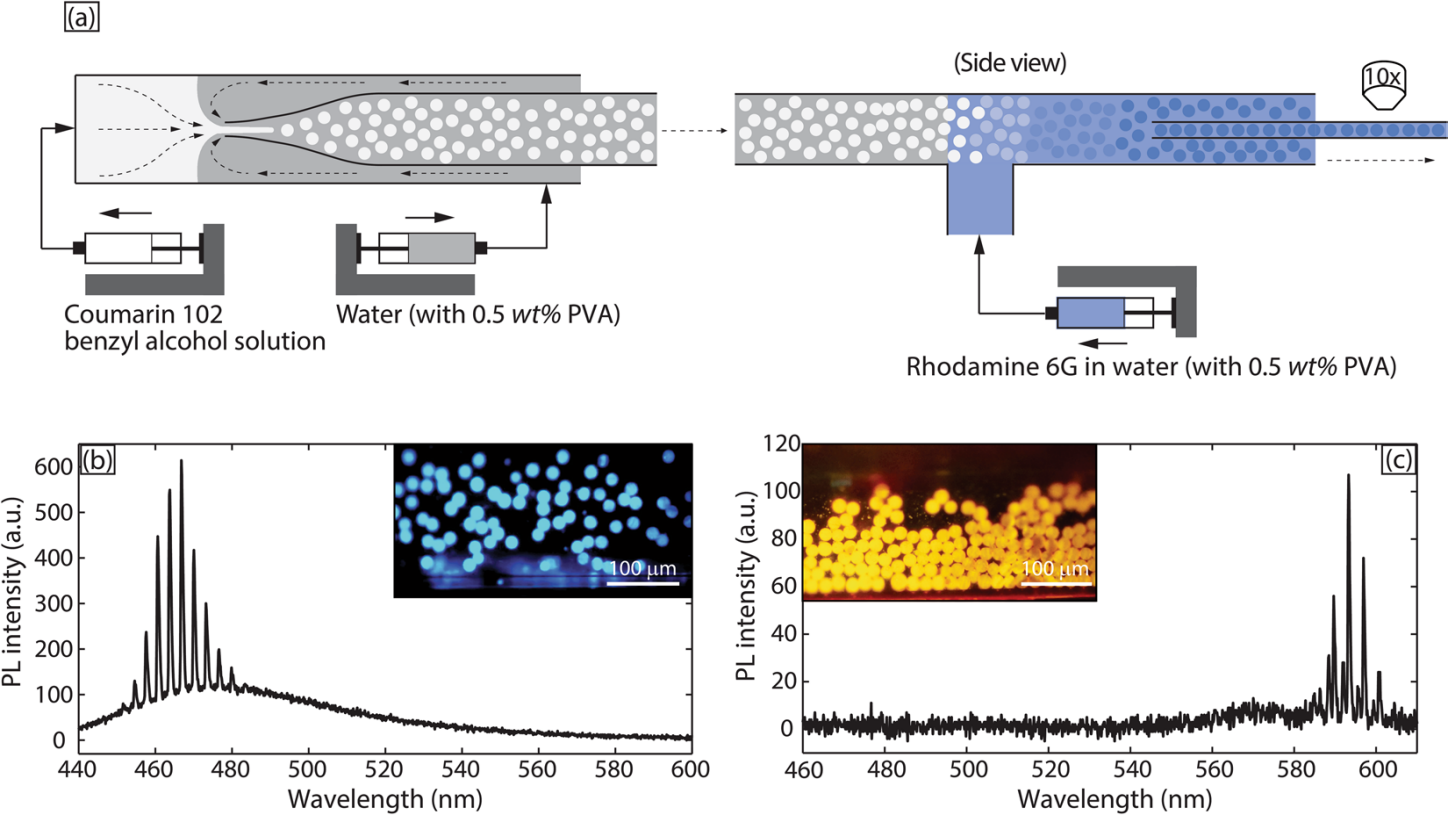
(**a**) A schematic of the method to introduce rhodamine 6 G into coumarin 102 loaded benzyl alcohol droplets. O/W_1_ emulsion droplets were generated in a flow-focusing glass capillary and directed into a T-junction, where W_2_ was introduced. The outlet of the T-junction was then directed into a high aspect ratio square capillary for single layer optical excitation and measurements. (**b**) A typical spectrum obtained from coumarin 102 loaded droplets where W_2_ composition was the same as W_1_. Inset: Stereomicroscopic image of the highly monodisperse emulsion droplets in a single layer excited by a pulsed pump laser beam. (**c**) A typical spectrum obtained from droplets loaded with both coumarin 102 and rhodamine 6 G where O consisted of 5 mM coumarin 102 in benzyl alcohol solution and W_2_ consisted of a 1.5 mM rhodamine 6 G aqueous solution with surfactant. Inset: Stereomicroscopic image of the highly monodisperse emulsion droplets in a single layer excited by a pulsed pump laser beam.

As shown in Fig. 1(a), monodisperse droplets of coumarin 102 (5 mM) in benzyl alcohol (refractive index, *n*_*D*_ = 1.54) were generated by a flow-focusing glass capillary device. The carrier fluid was an aqueous solution containing a small quantity (0.5 wt%) of polyvinyl alcohol (PVA) as surfactant (refractive index, *n*_*D*_ = 1.33). Benzyl alcohol was chosen as a solvent for coumarin 102 for two reasons: (i) its high refractive index contrast with water ensures efficient WGM-based light confinement at the boundary of the droplets^5,13^, and (ii) it is a good solvent for many laser dyes, including rhodamine 6 G, which allows rapid diffusion of rhodamine 6 G from aqueous continuous phase into coumarin 102-loaded droplets. Rhodamine 6 G is a widely used laser dye with a quantum yield of 0.95^24^; it has an absorption peak around 530 nm (minimum absorption at the pump pulse wavelength 400 nm), thus providing sufficient spectral overlap with coumarin 102 for energy transfer. The droplets were then fed into a T-junction, as shown in Fig. 1. Figure 1(b) shows the results of a control experiment in which an aqueous stream containing 0.5 wt% PVA was introduced into the T-junction; the droplets show blue emission, as expected with the use of coumarin 102. Figure 1(c) shows the results of an experiment in which an aqueous rhodamine 6 G (1.5 mM) solution with 0.5 wt% PVA was introduced online via the same T-junction, to switch the color of the droplet lasers from blue to orange. In both cases, the droplets were then directed into a high aspect ratio 300 × 30 μm (width × height) square glass capillary which allowed a monolayer flow of droplets (droplet diameter ~14 μm in the case of Fig. 1(b) and ~17 μm in the case of Fig. 1(c). In both cases, the standard deviations of droplet diameters were <5%). The droplets were excited by a Ti:Sapphire pump laser system with a repetition rate of 1 kHz and a pulse duration of 100 fs at an excitation wavelength of 400 nm. The excitation spot size was ~600 μm in diameter and droplet speed in the high aspect ratio square capillary was between 5–30 μm/s, depending on size of the droplets. Figure 1(b) shows a typical lasing spectrum obtained from the coumarin 102-loaded droplets, without addition of rhodamine 6 G, and the corresponding spectrum for the case of rhodamine 6 G addition downstream is shown in Fig. 1(c). The insets of Fig. 1(b,c) are stereomicroscopic images of the excited droplets, obtained by blocking out the scattered laser light through a dichroic filter. The black background in the inset of Fig. 1(c), despite the presence of rhodamine 6 G in the aqueous phase indicates minimal rhodamine 6 G emission in the absence of coumarin 102, which is located within the benzyl alcohol droplets. The spectral narrowing and appearance of equally spaced lasing peaks from rhodamine 6 G emission, together with the disappearance of coumarin 102 emission in Fig. 1(c) strongly indicate a highly efficient energy transfer process.

**Figure 2:**
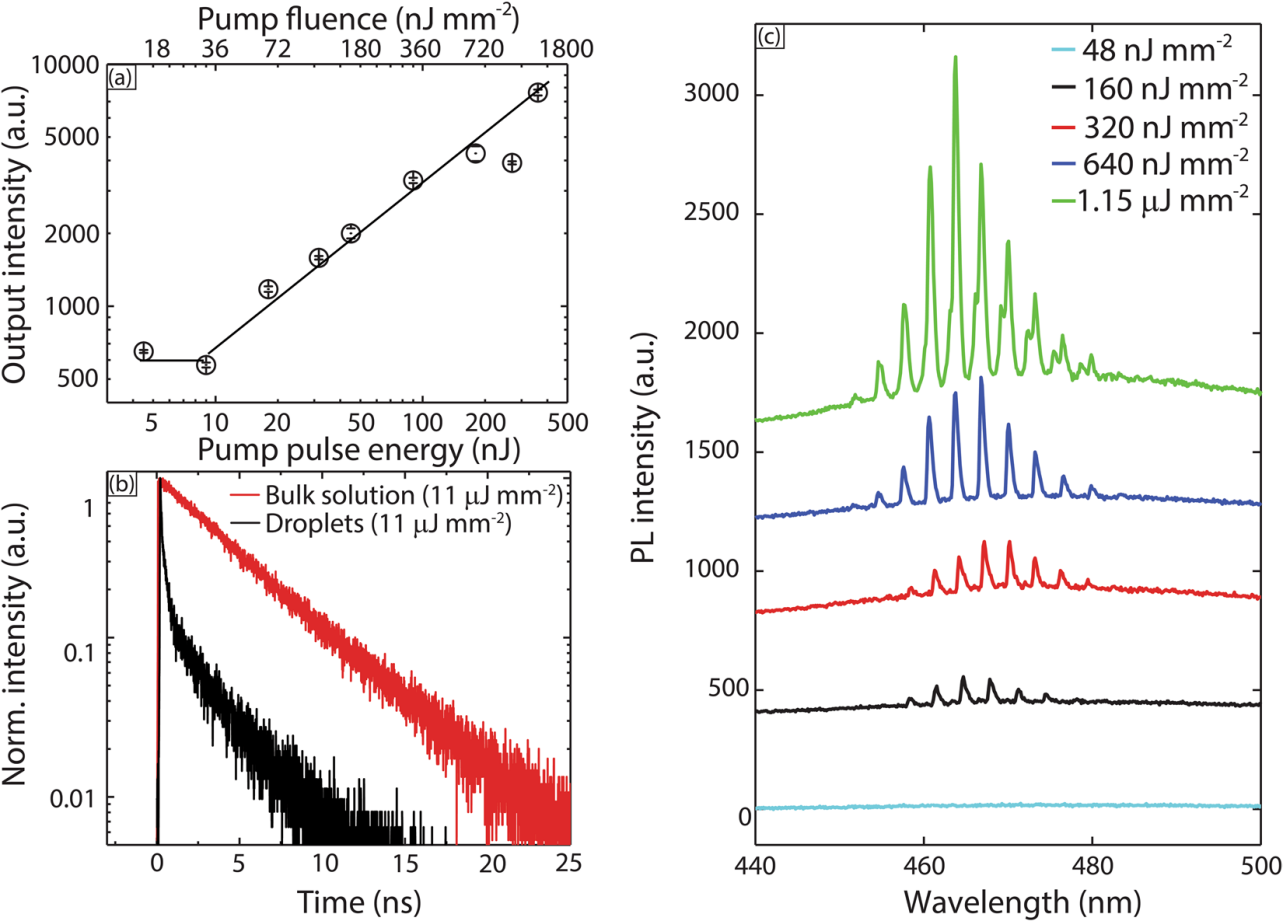
(**a**) Nonlinear dependence of lasing emission intensity on pump pulse energy (**b**) Fluorescence decay profile of spontaneous emission (bulk PL) and lasing (droplets). (**c**) Lasing spectra from coumarin 102 loaded droplets of ~14 μm diameter at pump fluence below and above lasing threshold. Spectra are staggered vertically for clarity.

Figure 2(a) shows the output intensities as a function of pump pulse energy for 22 μm (diameter) droplets containing 5 mM solutions of coumarin 102 in benzyl alcohol; the points are averaged from 10 sets of data. The observed threshold pulse intensity was about 36 nJ/mm^2^. Emission lifetime data obtained from time-correlated single photon counting (TCSPC) further validated the occurrence of lasing (Fig. 2(b)). Fitting the data with an exponential decay function revealed a lifetime ~100 ps (close to the Instrument Response Function (IRF) of the TCSPC) for the droplets, and a lifetime ~4.6 ns for photoluminescence from the bulk solution inside the capillary. The much shorter lifetime of the droplets is consistent with the occurrence of stimulated emission, which is a much faster process compared to spontaneous emission^16^. These results thus validate the occurrence of a blue laser (460–480 nm) with flowing droplets loaded with coumarin 102 (donor).

**Figure 3:**
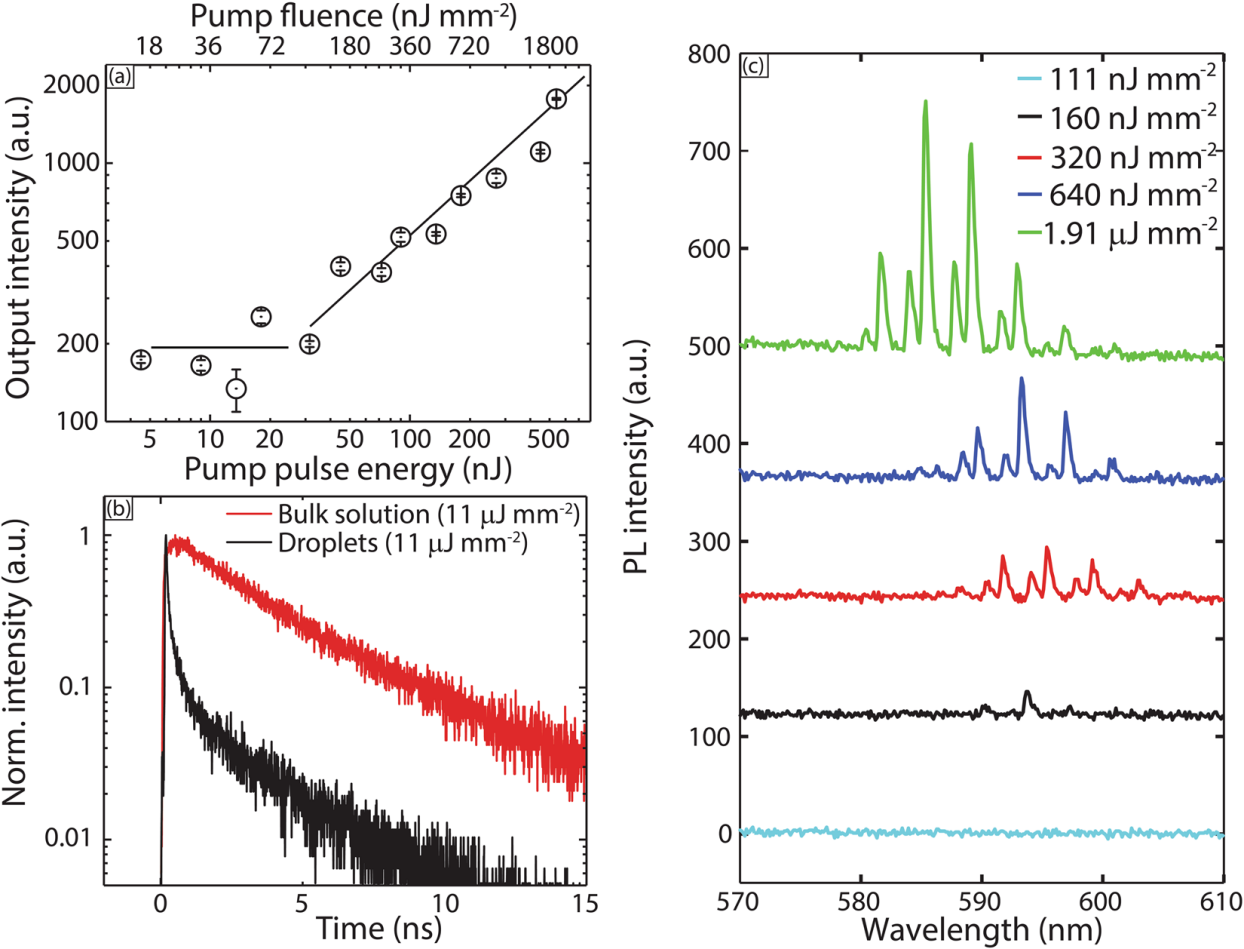
(**a**) Nonlinear dependence of lasing emission intensity on pump pulse energy (**b**) Fluorescence decay profile of spontaneous emission (bulk PL) and lasing (droplets). (**c**) Lasing spectra from coumarin 102 loaded droplets, after addition of rhodamine 6 G, of ~17 μm diameter at pump fluence below and above lasing threshold. Spectra are staggered vertically for clarity.

Next, we studied color switching to an orange laser (580–600 nm) achieved through addition of rhodamine 6 G downstream, followed by the transport of rhodamine 6 G from the aqueous phase into the coumarin 102-loaded benzyl alcohol droplets to achieve chemical equilibrium. This transport occurs very rapidly; the estimated diffusion time for rhodamine 6 G into 20 μm coumarin 102-loaded droplets is ~0.4 s. Therefore, in our experiments, the spectra collected immediately after rhodamine 6 G addition (i.e. as soon as the droplets exited the T-junction into the square capillary), showed only emission from rhodamine 6 G. Threshold analysis (Fig. 3(a)) reveals a lasing threshold below ~180 nJ/mm^2^ (for 17 μm droplets). TCSPC lifetime data (Fig. 3(b)) indicates a lasing lifetime ~90 ps (close to the Instrument Response Function (IRF) of the TCSPC) of the droplets and a bulk solution photoluminescence lifetime ~3.7 ns. Spectra obtained at different pump fluences are presented in Fig. 3(c).

**Figure 4:**
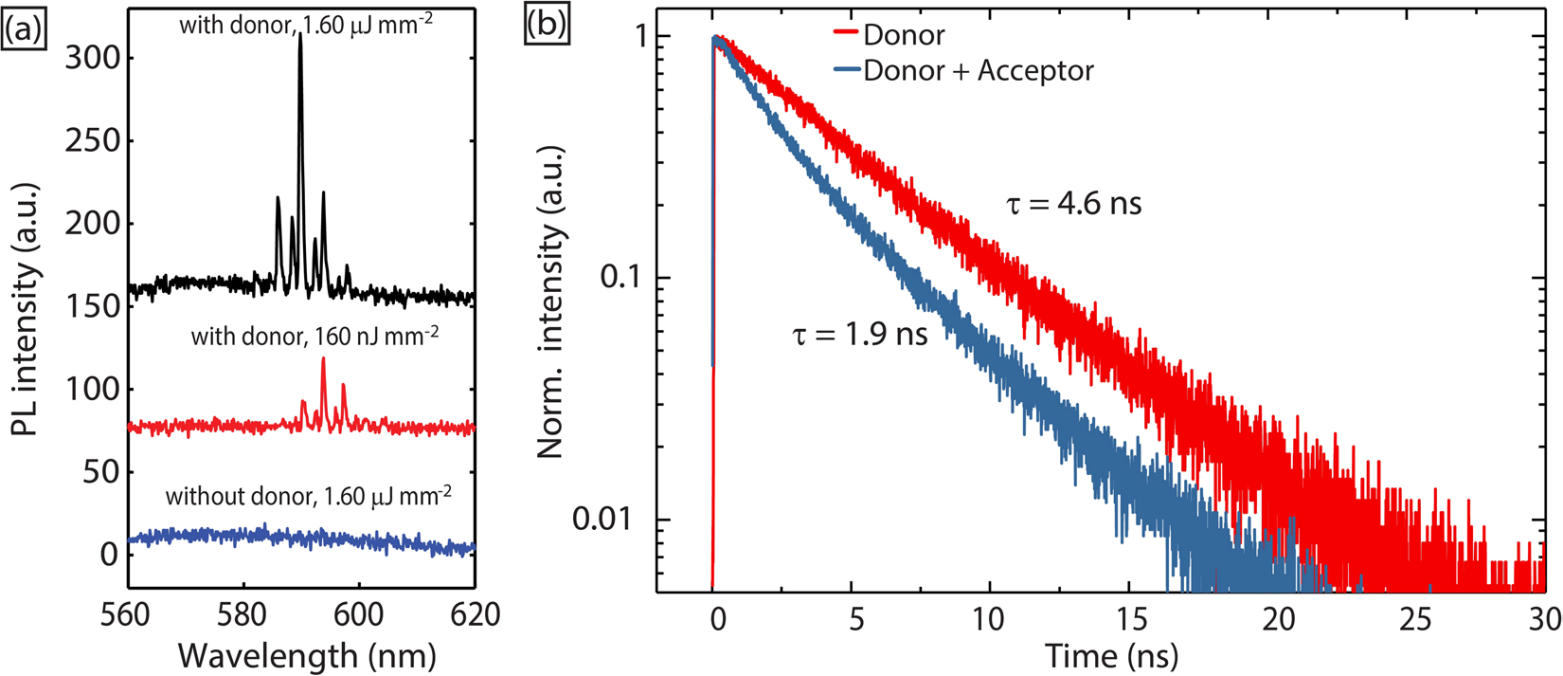
(**a**) Droplet spectra comparison: lasing with coumarin 102 (donor) and featureless emission without coumarin 102. (**b**) Fluorescence decay profile of bulk donor and model case (donor with acceptor).

We conducted a control experiment without coumarin 102 in the droplets to confirm that the pumping mechanism for the orange laser was indeed energy transfer based. Within the range of pump fluences used in the experiments above, no lasing could be observed. As shown in Fig. 4(a), without coumarin 102, there was only a weak featureless photoluminescence signal from benzyl alcohol droplets after rhodamine 6 G addition, which is consistent with the low absorption cross section for rhodamine 6 G at 400 nm. In contrast, even at pump fluences 10 times lower, lasing peaks emerged in the case with coumarin 102 loaded in the benzyl alcohol droplets (Fig. 4(a)). There are two possible energy transfer mechanisms in the droplet system - a short-ranged non-radiative Förster transfer i.e. FRET^3,16,17,22,23^, or cavity-assisted radiative energy transfer i.e. CARET, where photons emitted by the donor are stored in the WGM cavity and efficiently reabsorbed by the acceptor^19,23,25^. As FRET introduces a non-radiative pathway into the system, we would expect a shorter lifetime of the donor in the case of FRET^26^. However, the successful switching of the laser colors in our system means no emission from the donor molecule in our system. Thus, there is no emission from coumarin 102 at all pump fluences used in our experiments, from 18 nJ/mm^2^ to 1.8 μJ/mm^2^, making it impossible to directly compare the lifetime of the donor with and without the acceptor. Indeed, the *absence* of coumarin emission points strongly to FRET as the energy transfer mechanism, since it is expected that coumarin emission should be observable for CARET. Now, the observability of coumarin emission in CARET is contingent on two conditions: (i) that CARET is inefficient and (ii) the remaining coumarin photons are effectively scattered into the detector. However, in the event that (i) and/or (ii) are not satisfied, the emission spectrum analysis is not conclusive. We therefore did a lifetime measurement experiment in the absence of a cavity to definitively rule out CARET in favor of FRET, as discussed below.

Since both the donor and acceptor are distributed homogeneously in solution, we can determine the average donor-acceptor distance based on their concentrations^17^. We used the UV-Vis absorbance of rhodamine 6 G solutions in benzyl alcohol to determine the equilibrium concentration of rhodamine 6 G in coumarin 102-loaded (5 mM) benzyl alcohol droplets to be 9 mM (See Experimental Section for details). The average distance between the two dyes can be estimated to be 6.1 nm by assuming a homogeneous distribution of dye molecules in solution^17^, which is within the Förster distance range of most dye-dye pairs (2–10 nm)^27^. When a bulk benzyl alcohol solution containing 5 mM of coumarin 102 and 9 mM of rhodamine 6 G was illuminated by the 400 nm fs pulsed laser, there was no emission from coumarin 102, while a much stronger PL signal was observed from rhodamine 6 G, consistent with the case of the microfluidic droplets. This indicates sufficient energy transfer efficiency even in the absence of a microcavity. Thus, we infer that cavity-assisted radiative energy transfer is likely not the dominant energy transfer mechanism. Now, as the Förster transfer efficiency between a single donor and a single acceptor is highly dependent on the donor-acceptor distance^16^, we then increased the relative concentration of the donor to the acceptor, while maintaining the average distance between the donor and acceptor constant, so as to ensure sufficient emission from the donor for a lifetime measurement. In similar fashion to the above measurement, the donor (15 mM) and acceptor (0.2 mM) were dissolved in benzyl alcohol (estimated donor-acceptor distance of 5.9 nm) and placed in a UV cuvette for lifetime measurement. TCSPC lifetime data (Fig. 4(b)) revealed a lifetime of ~1.9 n*s* for coumarin 102, which is ~2.5× smaller than the coumarin 102 lifetime (~4.6 ns) in the absence of rhodamine 6 G, confirming that FRET is the operating mechanism for energy transfer in our system. To confirm that the reduction of coumarin lifetime is not due to self-quenching at high concentration (15 mM), we did another lifetime measurement for coumarin 102 alone at 15 mM, which was found to be ~5.2 ns.

**Figure 5:**
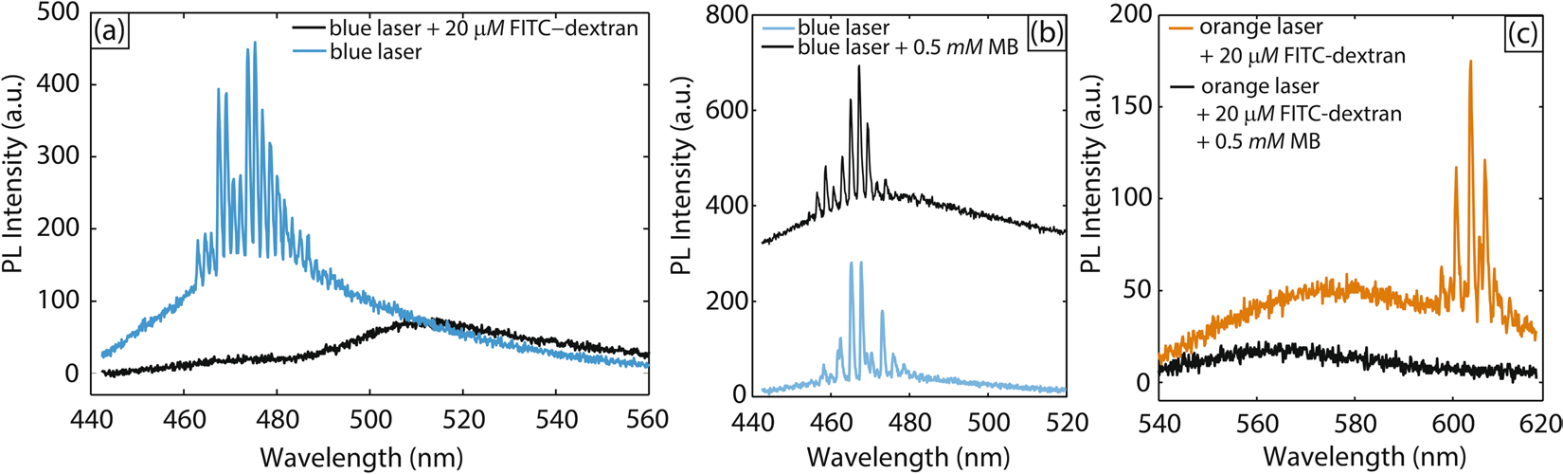
(**a**) Spectra collected from excitation of microfluidic droplets in two different conditions at a pump fluence = 640 nJ/mm^2^: (i) blue laser droplets surrounded by an aqueous solution with surfactant, and (ii) blue laser droplets surrounded by a 20 μM FITC-dextran aqueous solution with surfactant. (**b**) Spectra collected from excitation of microfluidic droplets in two different conditions at a pump fluence = 640 nJ/mm^2^: (i) blue laser droplets surrounded by an aqueous solution with surfactant, and (ii) blue laser droplets surrounded by a 0.5 mM MB aqueous solution with surfactant. Spectra were shifted upwards by an arbitrary value for clarity purpose. (**c**) Spectra collected from excitation of microfluidic droplets in two different conditions at a pump fluence = 640 nJ/mm^2^: (i) orange laser droplets surrounded by a 20 μM FITC-dextran aqueous solution with surfactant, and (ii) orange laser droplets surrounded by a 20 μM FITC-dextran and 0.5 mM MB aqueous solution with surfactant.

Monitoring the changes in the laser output, such as intensity and spectral characteristics enables the detection of hard-to-distinguish small signals^28^. WGM resonators are ideal candidates for optical sensing of pressure, temperature, humidity, electric fields, and also for chemical/biological sensing^11^. In our recent work^15^, we have shown a proof-of-concept detection demonstration in which coumarin 102-loaded droplet lasers were completely shut off due to the presence of an absorbing molecule (fluorescein isothiocyanate-dextran conjugate) in their vicinity. The multi-color droplet laser platform introduced in this work now enables *sequential* detection of analytes. To demonstrate this, we choose fluorescein isothiocyanate-dextran conjugate (hereafter as FITC-dextran) and methylene blue (hereafter as MB) as model analytes. FITC-Dextran has an excitation maximum at 490 nm, which is located well within the emission range of the coumarin 102 blue laser. 20 μM FITC-Dextran aqueous solution with surfactant (0.5 wt% PVA) was introduced into the continuous phase after droplet generation but before laser color switching, using the same configuration shown in Fig. 1(a). We then excited these droplets with the 400 nm pulsed laser at a pump pulse energy of 640 nJ/mm^2^. As shown in Fig. 5(a), the WGM-modulated modes in the blue laser spectra were shut off and only PL remained in the fluorescence spectra, which is consistent with what we have demonstrated previously^15^. Next, 0.5 mM MB aqueous solution with surfactant (0.5 wt% PVA) was introduced to the continuous phase after droplet generation. We again excited these droplets with the 400 nm pulsed laser at a pump pulse energy of 640 nJ/mm^2^. MB absorbs in a visible light wavelength range from 550 nm to 700 nm with a maximum at 665 nm^29^, which falls in the emission range of our orange rhodamine 6 G lasers. However, MB has nearly zero absorption in the range of 350–500 nm^29^; therefore, as shown clearly in Fig. 5(b), without the addition of rhodamine 6 G into the droplet cavity, the blue laser (coumarin 102) had no apparent change in intensity or spectral characteristics in response to the addition of MB to its surrounding medium (continuous phase). Next, we checked whether the orange laser could detect MB in the presence of FITC-dextran. With the addition of rhodamine 6 G, i.e. lasing color switched to orange, we introduced an aqueous solution containing *both* 20 μM FITC-Dextran and 0.5 mM MB; in this case, the WGM modes in the orange laser spectra were shut off and only PL remained in the fluorescence spectra as shown in Fig. 5(c). Thus, the orange laser is capable of detecting MB. To demonstrate that our system was capable of *sequentially* detecting both FITC-dextran and MB, it was important to verify that the shutdown of the orange laser was not due to the presence of FITC-dextran. Therefore, we carried out another experiment where we introduced an aqueous solution containing 20 μM FITC-Dextran into the vicinity of the orange lasers after color switching. Figure 5(c) shows that the orange laser was not affected and the WGM-modulated modes were still present in the spectra. Hence, indeed, with the added capability of online lasing color switching within the same droplet cavity, we can conduct sequential detection of a series of analytes in the vicinity of these droplet lasers. To illustrate the detection scheme better, we have constructed a table (Table 1) to summarize all the possible outcomes.

**Table 1:** Detection scheme illustration.

Scenario	Blue laser	Orange laser
Coumarin 102	Yes	No
Coumarin 102 + BLQ*	No	No
Coumarin 102 + OLQ**	Yes	No
Coumarin 102 + R6G	No	Yes
Coumarin 102 + BLQ* + R6G	No	Yes
Coumarin 102 + R6G + OLQ**	No	No

*(BLQ): blue laser quencher, FITC-dextran here. **(OLQ): orange laser quencher, methylene blue here.

## Conclusions

We have demonstrated a multi-color dye laser platform using chemically open microfluidic droplet cavities via a non-radiative Förster transfer (FRET) process, where the time and place of color switching is precisely controlled without altering the cavity properties. This platform enables sequential detection of a series of analytes using the same droplet cavities. We have also conducted a proof-of-concept demonstration in which two model analytes – FITC-Dextran and MB were detected by the blue and orange droplet lasers respectively. This platform can be extended to a wide selection of water-soluble laser dyes, where an extended laser color switching spectral range can be achieved via sequential energy transfer. With smart choice of both dispersed and continuous phases, laser color switching can even be achieved by extracting certain dye molecules *out* from the droplet cavities, thus fully exploiting the chemical openness of flowing microfluidic droplets. There are several considerations that require special attention for potential applications of this method. For example, judicious choice of the donor/acceptor dye pair is the crucial determinant of the detection range, and choices for the acceptor are currently limited to water-soluble dyes. Other limitations of the current method include the use of an alcohol and high dye concentrations, which might restrict biological applications of the system. These limitations may, to some extent, be addressed by careful choice of the non-aqueous phase - a choice that spans a wide design space and can include ‘designer’ fluids such as ionic liquids with tunable physical properties^30^. In summary, we believe that our novel platform opens up new possibilities in optofluidic WGM sensing, where sequential detection of analytes is now made possible using chemically open droplet cavities.

## Experimental Section

### Materials

Poly(vinyl) alcohol (PVA) (M.W. − 67,000), coumarin 102 (dye content 99%), benzyl alcohol (99.5%), rhodamine 6 G (dye content ~95%), Fluorescein isothiocyanate – Dextran 500000-Conjugate were purchased from Sigma-Aldrich (Singapore) and used as received. Methylene Blue (high purity, biological stain) was purchased from Alfa Aesar and used as received. Ultrapure water (18.3 MΩ) obtained using a Millipore Milli-Q purification system was used to prepare aqueous PVA solution. Harvard PHD 22/2000 series syringe pumps were used to dispense fluids into the emulsion generator. Square and cylindrical glass capillaries of ID 1 mm and 0.7 mm respectively, and high aspect ratio square capillary (W × H = 0.3 × 0.03 mm) were purchased from Arte glass Associates Co. Ltd., Japan. Animal-Free natural polypropylene tee tube fitting with classic series barbs (T-junction) for 1.6 mm ID tubing was purchased from Value Plastics, Inc.

## Methods

### Droplet Generation

Benzyl alcohol in water droplets (O/W_1_ emulsions) were generated using a glass capillary microfluidic setup (Fig. 1(a), Please refer to Figure S1 in Supporting Information for details). The axisymmetric coaxial glass capillary flow-focusing device was assembled using a square and a round capillary with a tapered end^31–33^. The surface of the round capillary was hydrophilized by treatment with oxygen plasma (100 W) for 120 s. The dispersed phase (O) was a coumarin 102 (5 mM) in benzyl alcohol solution. The aqueous continuous phase (W) used was a 0.5 wt% PVA aqueous solution. W_1_ and O phases were infused from the two ends of the square capillary through the outer coaxial region using syringe pumps (Harvard PHD 22/2000 series) at flow rates of 3.5 and 0.5 µL/min respectively. The emulsion droplets were formed by hydrodynamic flow focusing through the nozzle of the inner round capillary.

### Rhodamine 6G Addition

After O/W_1_ emulsions were generated, they were directed into a T-junction, where another aqueous solution W_2_ was infused through the other end of the T-junction, using a syringe pump (Harvard PHD 22/2000 series) at a flow rate of 20 µL/min. The aqueous phase (W_2_) was one of the following: (i) a 0.5 wt% PVA aqueous solution for the blank case, or (ii) a rhodamine 6 G (1.5 mM) in 0.5 wt% PVA aqueous solution for the switch of laser color. The outlet from the T-junction was then directed to a high aspect ratio square capillary (W × H = 0.3 × 0.03 mm) to form single layer emulsion flows for optical measurements.

### Equilibrium Concentration of Rhodamine 6 G in Benzyl Alcohol

In a 10 ml glass vial, 1 ml of 5 mM coumarin 102 benzyl alcohol solution was carefully contacted with 5 ml of 1.5 mM rhodamine 6 G aqueous solution as two separate layers and left for 24 hours for equilibrium (diffusion characteristic time ~17 hours). Afterwards, the benzyl alcohol layer was separated out and used for UV-Vis absorbance measurement, using a Shimadzu UV-1800 UV-Vis spectrophotometer, to determine the concentration of rhodamine 6 G. We prepared various known concentrations (1.5–9 μM) of rhodamine 6 G in benzyl alcohol solutions to obtain the calibration curve. The equilibrium concentration was found to be 9 mM.

### Optical Excitation and Measurements

The optical setup is the same as our previous paper^15^. A schematic of the optical setup comprising the excitation and detection systems is depicted in Figure S2 in Supporting Information. The pump laser was a Ti:Sapphire laser system (Coherent) with a repetition rate of 1 kHz and a pulse duration of 100 fs at wavelength of 400 nm tuned by an optical parametric amplifier. The pump beam was coupled into a 10× microscope objective (numerical aperture *NA* = 0.3) and focused at the microfluidic emulsion generator, with a beam spot diameter ~ 600 µm. The laser signal was collected using an output multimode fiber and analyzed by a spectrometer (Princeton Imaging Spectrograph, resolution 0.13 nm). The pump power was measured by a power meter (FieldMate, Coherent) with a high-sensitivity optical sensor (OP-2 Vis, Coherent).

### TCSPC Measurement

Emission signals were routed from the above setup using a multimode output fiber and recorded by a time-correlated single photon counting (TCSPC) module (PicoQuant PicoHarp 300), at a count interval of 0.008 ns, with excitation at 400 nm and monitoring at 500 nm for coumarin 102 and 600 nm for rhodamine 6 G.

## Electronic supplementary material

Supporting Information

## Data Availability

The datasets generated during and analysed during the current study are available from the corresponding author on reasonable request.

## References

[1] Chen Y (2010). Optofluidic microcavities: Dye-lasers and biosensors. Biomicrofluidics.

[2] Duarte, F. J., Kelley, P., Hillman, L. W. & Liao, P. F. *Dye laser principles: with applications*. (Academic Press, 1990).

[3] Cerdán L (2012). FRET-assisted laser emission in colloidal suspensions of dye-doped latex nanoparticles. Nature Photonics.

[4] Godin J (2008). Microfluidics and photonics for Bio‐System‐on‐a‐Chip: A review of advancements in technology towards a microfluidic flow cytometry chip. Journal of biophotonics.

[5] Tang SK (2009). A multi-color fast-switching microfluidic droplet dye laser. Lab on a Chip.

[6] Duarte, F. J. *Tunable laser applications*. Vol. 150 (CRC press, 2008).

[7] Barnes, N. P. *et al*. Tunable Lasers Handbook. *Academic San Diego* 219–291 (1995).

[8] Lee W, Luo Y, Zhu Q, Fan X (2011). Versatile optofluidic ring resonator lasers based on microdroplets. Optics express.

[9] Kuehne AJ (2011). A switchable digital microfluidic droplet dye-laser. Lab on a Chip.

[10] Aubry G (2011). A multicolor microfluidic droplet dye laser with single mode emission. Applied Physics Letters.

[11] Reynolds, T. *et al*. Fluorescent and lasing whispering gallery mode microresonators for sensing applications. Laser & Photonics Reviews (2017).

[12] Tang SK, Derda R, Quan Q, Lončar M, Whitesides GM (2011). Continuously tunable microdroplet-laser in a microfluidic channel. Optics express.

[13] Chang, R. K. & Campillo, A. J. *Optical processes in microcavities*. Vol. 3 (World scientific, 1996).

[14] Tanyeri M, Perron R, Kennedy IM (2007). Lasing droplets in a microfabricated channel. Optics letters.

[15] Zheng L, Zhi M, Chan Y, Khan SA (2018). Embedding liquid lasers within or around aqueous microfluidic droplets. Lab on a Chip.

[16] Shopova SI (2007). Opto-fluidic ring resonator lasers based on highly efficient resonant energy transfer. Optics express.

[17] Sun Y, Shopova SI, Wu C-S, Arnold S, Fan X (2010). Bioinspired optofluidic FRET lasers via DNA scaffolds. Proceedings of the National Academy of Sciences.

[18] Zhang X, Lee W, Fan X (2012). Bio-switchable optofluidic lasers based on DNA Holliday junctions. Lab on a Chip.

[19] Kiraz A, Doğanay S, Kurt A, Demirel A (2007). Enhanced energy transfer in single glycerol/water microdroplets standing on a superhydrophobic surface. Chemical physics letters.

[20] Chen Q (2013). Highly sensitive fluorescent protein FRET detection using optofluidic lasers. Lab on a Chip.

[21] Aas M, Chen Q, Jonáš A, Kiraz A, Fan X (2016). Optofluidic FRET lasers and their applications in novel photonic devices and biochemical sensing. IEEE Journal of Selected Topics in Quantum Electronics.

[22] Chen Q, Kiraz A, Fan X (2016). Optofluidic FRET lasers using aqueous quantum dots as donors. Lab on a Chip.

[23] Özelci E, Aas M, Jonáš A, Kiraz A (2014). Optofluidic FRET microlasers based on surface-supported liquid microdroplets. Laser Physics Letters.

[24] Kubin RF, Fletcher AN (1982). Fluorescence quantum yields of some rhodamine dyes. Journal of Luminescence.

[25] Arnold S, Folan L (1989). Energy transfer and the photon lifetime within an aerosol particle. Optics letters.

[26] Selvin PR (2000). The renaissance of fluorescence resonance energy transfer. Nature Structural & Molecular Biology.

[27] Lakowicz Joseph R. (1999). Fluorescence Sensing. Principles of Fluorescence Spectroscopy.

[28] Fan X, Yun S-H (2014). The potential of optofluidic biolasers. Nature methods.

[29] Melgoza D, Hernandez-Ramirez A, Peralta-Hernandez J (2009). Comparative efficiencies of the decolourisation of Methylene Blue using Fenton’s and photo-Fenton’s reactions. Photochemical & Photobiological Sciences.

[30] Barikbin Z (2010). Ionic liquid-based compound droplet microfluidics for ‘on-drop’separations and sensing. Lab on a Chip.

[31] Leon RAL, Wan WY, Badruddoza AZM, Hatton TA, Khan SA (2014). Simultaneous Spherical Crystallization and Co-Formulation of Drug(s) and Excipient from Microfluidic Double Emulsions. Crystal Growth & Design.

[32] Toldy AI (2012). Spherical Crystallization of Glycine from Monodisperse Microfluidic Emulsions. Crystal Growth & Design.

[33] Utada A (2005). Monodisperse double emulsions generated from a microcapillary device. Science.

