# Supplementary material for: Multi-color lasing in chemically open droplet cavities
Source: Sci Rep. 2018 Sep 20;8:14088. doi: 10.1038/s41598-018-32596-8 (PMC6147796; doi:10.1038/s41598-018-32596-8)
Supplement: Supplementary file 1 — Supporting Information [file 41598_2018_32596_MOESM1_ESM.docx]

Supporting Information

Multi-color lasing in chemically open droplet cavities

Lu Zheng^1^, Min Zhi^2^, Yinthai Chan^2a*^ and Saif A. Khan^1b*^

^1^Department of Chemical and Biomolecular Engineering, 3 Engineering Drive 3, National University of Singapore, Singapore 117582, Singapore

^2^ Department of Chemistry, 3 Science Drive 3, National University of Singapore, Singapore 117543, Singapore

^*^Corresponding Authors. ^a^ Email: chmchany@nus.edu.sg

^b^ Email: saifkhan@nus.edu.sg

KEYWORDS: Chemically open cavities, FRET lasers, Multi-color lasers, Droplet microfluidics, Whispering Gallery Mode, Optical resonances


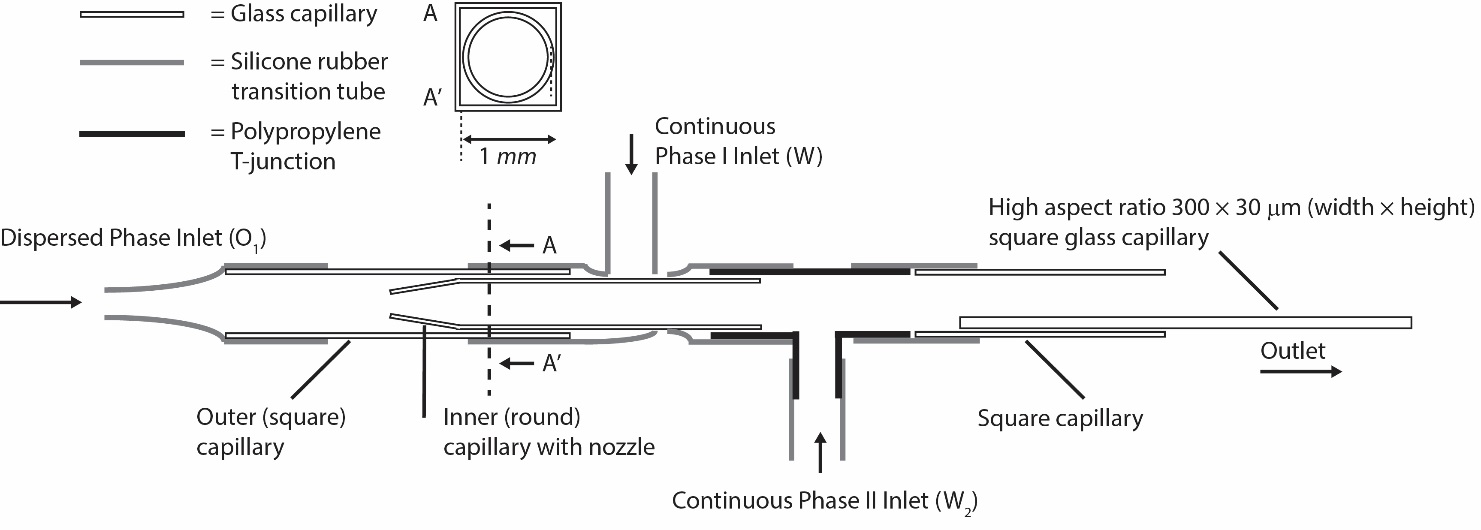


**Figure S1.** A schematic of the device used for emulsion generation and rhodamine addition


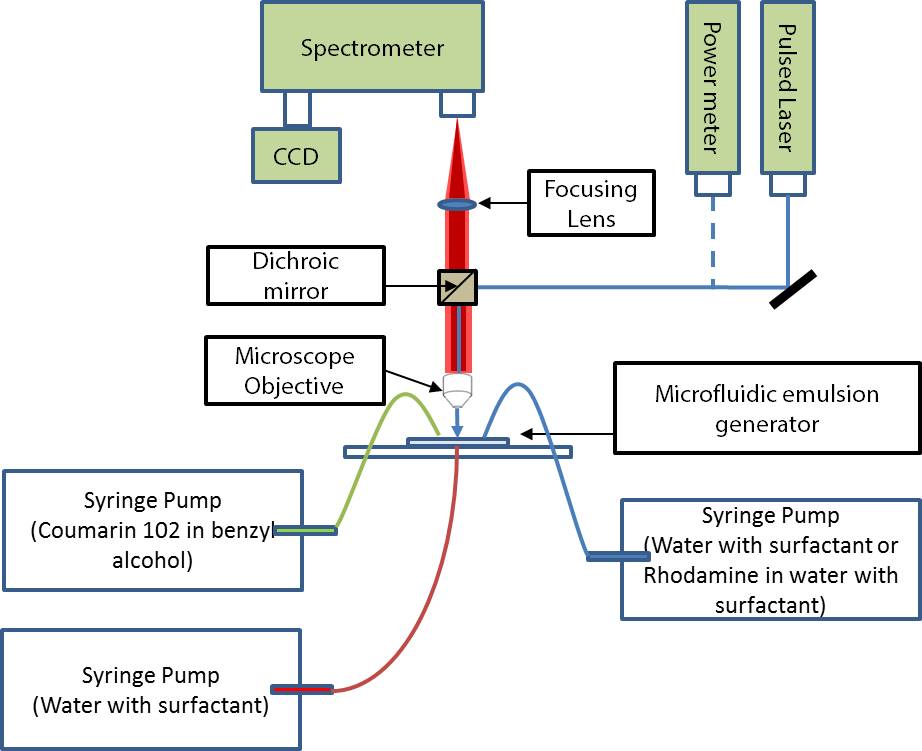


**Figure S2.** A schematic of experimental setup for optical resonance experiments
